# Exploring 6-Azaindole and 7-Azaindole Rings for Developing Cannabinoid Receptor 1 Allosteric Modulators

**DOI:** 10.1089/can.2018.0046

**Published:** 2018-12-10

**Authors:** Sri Sujana Immadi, Rachel Dopart, Zhixing Wu, Boqiao Fu, Debra A. Kendall, Dai Lu

**Affiliations:** ^1^ Department of Pharmaceutical Sciences, Rangel College of Pharmacy, Health Science Center, Texas A&M University, Kingsville, Texas.; ^2^ Department of Pharmaceutical Sciences, University of Connecticut, Storrs, Connecticut.

**Keywords:** allosteric modulators, azaindole, bioisostere, cannabinoid, CB_1_ receptor

## Abstract

**Introduction and Objective:** Org27569 is a prototypical allosteric modulator of the cannabinoid receptor 1 (CB_1_). It belongs to the indole-2-carboxamide scaffold and has been intensively investigated in pharmacology and in structure–activity relationship (SAR) studies. Although azaindoles are rare in natural products and differ only by the presence of an extra ring nitrogen, they were demonstrated as valuable bioisosteres in many pharmacologically important molecules. To extend the SAR investigation of the indole-2-carboxamide class of CB_1_ allosteric modulators, azaindole (pyrrolopyridine) rings were used to replace the indole ring of Org27569 analogs to explore the potential of azaindole-2-carboxamides as CB_1_ allosteric modulators. Using 6- and 7-azaindole in lieu of the indole moiety within this class of CB_1_ allosteric modulators indeed improved the aqueous solubility.

**Materials and Methods:** We synthesized 6- and 7-azaindole-2-carboxamides and their indole-2-carboxamide counterparts. The molecules were evaluated by [^3^H]CP55,940 binding and [^35^S]GTPγS binding assays for their allosteric modulation of the CB_1_ receptor.

**Results:** The 7-azaindole-2-carboxamides lost the ability to bind to the CB_1_ receptor. The 6-azaindole-2-carboxamides (e.g., 3c and 3d) showed markedly reduced binding affinities to the CB_1_ receptor in comparison with their indole-2-carboxamide counterparts. However, they behaved similarly as indole-2-carboxamides in potentiating the orthosteric agonist binding and inhibiting the orthosteric agonist-induced G-protein coupling. The results indicated that some azaindole scaffolds (e.g., 6-azaindole) are worth further exploration, whereas the 7-azaindole ring is not a viable bioisostere of the indole ring in the Org27569 class of CB_1_ allosteric modulators.

## Introduction

The cannabinoid receptor 1 (CB_1_) is a G-protein-coupled receptor and has been recognized as a promising target for the treatment of many disorders, including pain, inflammation, metabolic syndromes, and neurodegenerative diseases.^1^ However, the CB_1_ receptor has been challenging as a druggable target because of central nervous system side effects associated with drug candidates that bind to the orthosteric site where the endogenous cannabinoids bind. Therefore, recent efforts have focused on developing allosteric modulators that target CB_1_ at sites topographically distinct from the orthosteric sites.^2^ Several small molecules have been revealed as CB_1_ allosteric modulators over the last 10 years.^2–4^

Org27569 is an indole-2-carboxamide and a prototypical CB_1_ allosteric modulator that has been intensively investigated.^5–7^ Following its discovery, several structure–activity relationship studies have addressed the key requirements to maintain or improve allosteric modulation effects.^8^ Most of the structural optimization of Org27569 was based on the indole moiety except two cases, in which benzofuran and benzimidazole rings were used in lieu of the indole ring. The binding affinities of the benzofuran analogs of Org27569 were significantly reduced, although the binding cooperativities with the orthosteric agonist were markedly enhanced.^9^ Similarly, the binding affinities of benzimidazole analogs of Org27569 were abolished, while allosteric modulation on agonist-induced GTPγS binding was maintained.^10^

In recent years, azaindoles (pyrrolopyridine) have gained significant attention as the bioisosteres of the indole ring due to their ability to facilitate pharmaceutical optimization, such as increasing solubility, reducing lipophilicity, enhancing target binding, and improving ADME as well as toxicology properties.^11–15^

The fusion of a pyrimidine ring and a pyrrole ring can provide a pyrrolopyridine structure having four isomers (i.e., 4-, 5-, 6-, and 7-azaindoles). Among them, the 7-azaindoles have been demonstrated with luminescent and fluorescent properties, which can render the molecule containing the 7-azaindole moiety with some chromophoric functions.^11,12^ In the synthesis of a group of cannabinoid receptor agonists from the known cannabinoid ligand JWH-018, it was demonstrated that bioisosteric replacement of the indole ring with the azaindole moieties (e.g., 5-, 6-, and 7-azaindoles) substantially improved the physicochemical properties of the resultant compounds.^16^ In addition, bioisosteric replacement of the indole ring with an azaindole moiety can enhance the drug–target interaction by formation of an extra hydrogen bond and significantly increase the pharmacological effects.^17,18^

**FIG. 1.**
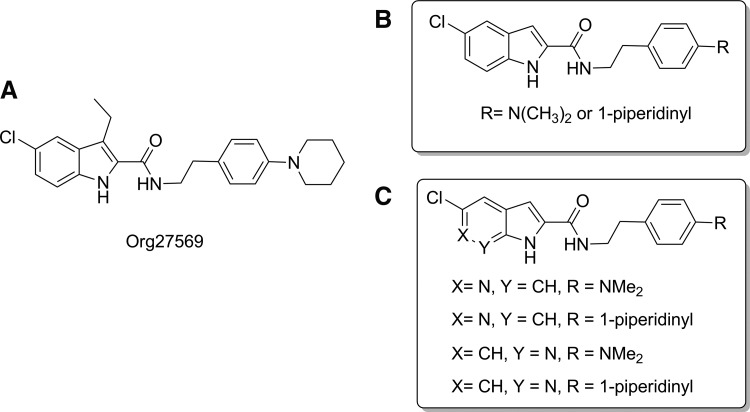
Prototypical CB_1_ allosteric modulator Org27569 **(A)**, referenced indole-2-carboxamide analogs of Org27569 **(B)**, and designed 6- and 7-azaindole-2-carboxamides **(C)**. CB_1_, cannabinoid receptor 1.

In addition to the frequently used 5-, 6-, and 7-azaindoles, the 4-azaindoles also was reported as a viable bioisosteric replacement of indole.^19^ With the goal of increasing aqueous solubility and developing a new scaffold, we designed and synthesized 6-azaindole-2-carboxamides (3c, 3d) and 7-azaindole-2-carboxamides (9a, 9b) to compare with their indole-2-carboxamide counterparts (3a and 3b) to explore the possibility of developing CB_1_ allosteric modulators. We used dimethylaminophenyl ethylamine and piperidinylphenyl ethylamine, which have been shown in the previous series of indole-2-carboxamides to improve function^8,20,21^ and to synthesize the target 6- and 7-azaindole-2-carboxamides shown in Figure 1C. The aqueous solubility of some synthesized compounds were assessed by measurement of their thermodynamic solubility because kinetic solubility data frequently overestimates solubility compared to thermodynamic solubility.^22^

## Materials and Methods

### Chemistry

The synthesis of reference indole-2-carboxamides 3a and 3b was carried out by coupling of commercially available 5-chloro-indole-2-carboxylic acid with commercially available 4-(2-aminoethyl)-*N*,*N*-dimethylaniline (2a) and 2-(4-(piperidin-1-yl)phenyl)ethanamine (2b), respectively, through the catalysis of 4-(4,6-Dimethoxy-1,3,5-triazin-2-yl)-4-methylmorpholinium chloride (DMTMM) and *N*-methyl morpholine (NMM) in THF. Similarly, 6-azaindole-2-carboxamides 3c and 3d were synthesized from 5-chloro-6-azaindole-2-carboxylic acid. To synthesize the 7-azaindole-2-carboxamides 9a and 9b, the required intermediate methyl 5-chloro-7-azaindole-2-carboxylate 7 was obtained through Hemetsberger–Knittel indole synthesis with a protocol previously optimized by us.^20^ Hydrolysis of the 7-azaindole-2-carboxylate 7 provided the essential intermediate 5-chloro-7-azaindole-2-carboxylic acid 8, which was coupled with 2a and 2b, respectively, to yield the target compounds 9a and 9b. The experimental details for the synthesis of compounds 3a–d, 6–8, and 9a–9b can be found in the Supplementary Data.

### Receptor expression and membrane preparation

Human embryonic kidney 293T (HEK293T) cells were grown and seeded in Dulbecco's modified Eagle's medium with 3.5 mg/mL glucose and 10% fetal bovine serum at 37°C and 5% carbon dioxide. To express the CB_1_ receptors, HEK293T cells were seeded at 1,000,000 cells/100-mm plate. The following day, cells were transfected via the calcium phosphate method with ∼10 μg of human CB_1_ receptor cloned into pcDNA3.1^23^ Membranes of the transfected cells were prepared as previously described 21 h after transfection.^24^

### Equilibrium binding assay

Approximately 3 μg of membrane preparation expressing CB_1_ was incubated with nine concentrations of allosteric modulator (1 nM-10 μM). The radiolabeled tracer [^3^H]CP55,940 (150.2 Ci/mmol; Perkin Elmer), an orthosteric agonist of CB_1_, was added at 0.5 nM. Nonspecific binding was established by treating with a high concentration of unlabeled CP55,940 (10 μM; Tocris). The membranes were incubated at 30°C for 60 min, and the reaction was terminated with the addition of 300 μL of Tris-Mg^2+^-EDTA (TME) buffer with 5% bovine serum albumin (BSA). The mixture was harvested by filtration through a Brandel cell harvester with Whatman GF/C filter paper. Liquid scintillation counting was used to measure radioactivity.

### GTPγS binding evaluation

To assess the impact of 3c and 3d on G-protein coupling of CB_1_, GTPγS assays were performed essentially as described previously.^20^ Membranes of CB_1_ expressing cells were prepared, and 8 μg of membrane preparation was incubated with 0.1 μM of CP55,940 plus or minus the allosteric modulator, allosteric modulator alone, or 1 μM SR141716A alone, and 0.1 nM [^35^S]GTPγS (1250 Ci/mmol; PerkinElmer Life Sciences, Boston, MA), 10 μM GDP (Sigma, St. Louis, MO), and 0.1% (w/v) BSA. GTPγS binding assay buffer (50 mM Tris-HCl, pH 7.4, 3 mM MgCl_2_, 0.2 mM EGTA, and 100 mM NaCl) was added to 200 μL. The membranes were incubated for 1 h at 30°C. To determine nonspecific binding, 10 μM unlabeled GTPγS (Sigma) was used. To determine basal activity, membrane preparations were treated with vehicle (dimethylsulfoxide or DMSO) alone. Termination of the reaction was achieved through filtration using Whatman GF/C filter papers and washing with cold TME buffer. Bound radioactivity was measured by liquid scintillation counting.

### Thermodynamic solubility assessment

The thermodynamic solubility of selected compounds were tested according to previously reported methods.^16,22^ In brief, stock solution of test compound (1 mg/mL) in acetonitrile was prepared and then diluted with mobile phase to prepare the calibration standards in the concentration range of 0.1–50 μg/mL. Following this, 2 mg of test compound powder was suspended in 2 mL of phosphate-buffered saline (PBS; 10 mM) at pH 7.4 and 37°C. The suspension was stirred at 37°C with 800 rpm stirring for 24 h. After completion of the incubation, the saturated suspension was filtered through a syringe polytetrafluoroethylene filter (0.45 μm), which has no absorption for the compounds. 200 μL of the filtrate was diluted with 800 μL acetonitrile immediately to a final volume of 1 mL. Subsequently, 20 μL of this solution was used for the high-performance liquid chromatography (HPLC) analysis, which was analyzed on Shimadzu HPLC (LC model: LC20AB, UV/vis detector: SPD20A; Autosampler: SIL-20A HT) with a Phenomenex reverse phase C18 column (250×3 mm, Luna 5 μm C18 100Å). The HPLC analysis was carried out by using the mixture of acetonitrile and water (60:40 v/v) as the mobile phase with a flow rate of 1 mL/min. The signals were detected at UV wavelength 254 nm. At this wavelength, each peak's area under the curve was measured and used for the quantitative evaluation.

## Results and Discussion

**FIG. 2.**
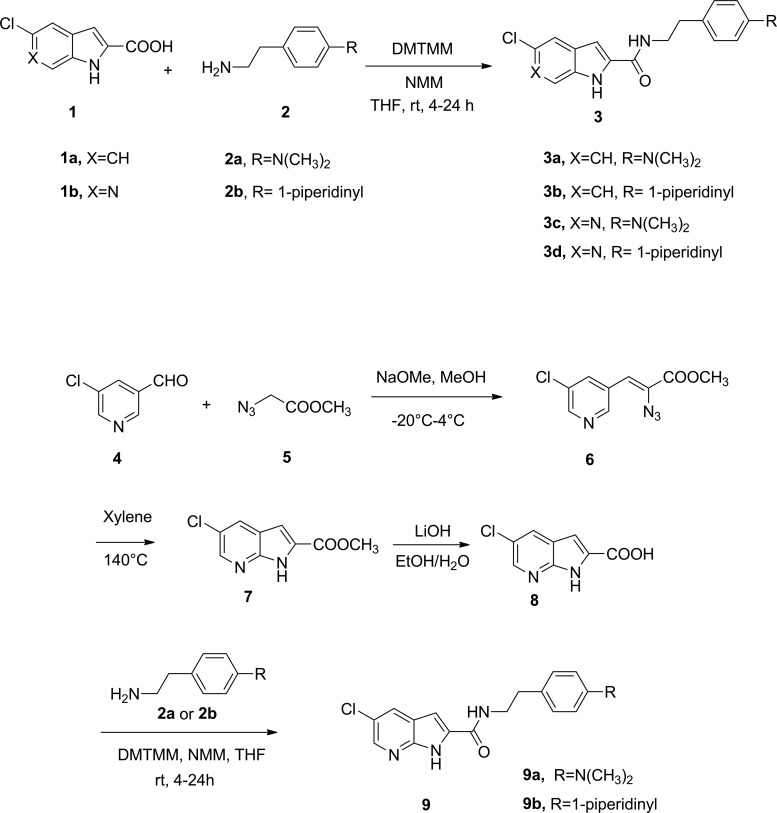
Synthesis of indole- and azaindole-2-carboxamides. DMTMM, 4-(4,6-Dimethoxy-1,3,5-triazin-2-yl)-4-methylmorpholinium chloride; NMM, *N*-methyl morpholine; THF, tetrahydrofuran.

To synthesize the target azaindole-2-carboxamides, it requires corresponding azaindole-2-carboxylic acids (i.e., 1b and 8) as the key building blocks (Fig. 2). To access these azaindole-2-carboxylic acids, we used the Hemetsberger–Knittel indole synthesis, which involves Knoevenagel condensation between a pyridinecarbaldehyde and the azido acetate 5.^20^ However, the condensation between 5-chloro-pyridine-3-carbaldehyde and the azido acetate 5 failed to provide the desired Knoevenagel product. In this investigation, the 6-azaindole-2-carboxylic acid 1b was obtained from a commercial source (Achemtek, Worcester, MA). In contrast, the condensation between 2-chloro-pyridine-4-carbaldehyde (4) and the azido acetate 5 successfully produced the corresponding Knoevenagel product 6, from which the required 7-azaindole-2-carboxylic acid (8) can be easily obtained. Unlike the indole-2-caboxylic acids, the coupling of the azaindole-2-carboxylic acids 1b and 8 with corresponding amines under the catalysis of HOBT or BOP and Hünig's base leads to low yields of the amide 3c-d and 9a-b. In contrast, catalyzing the coupling reaction by DMTMM and NMM generally provided the corresponding amides in good yields. The synthesized compounds were assessed by equilibrium binding assay,^25,26^ which identifies two important parameters for initial characterization of allostery: K_B_, the equilibrium dissociation constant that defines the affinity of an allosteric modulator for its receptor; and α, the cooperativity factor, which defines the magnitude and direction of impact that the allosteric modulator and orthosteric ligand have on each other when both occupy the receptor. At the receptor binding level, when α is >1, the ligand is a positive allosteric modulator, whereas when α is <1, the ligand is a negative allosteric modulator. Accordingly, when α is equal to 1, it indicates no allosteric modulation on orthosteric ligand binding. The K_B_ and α values of the synthesized compounds are presented in Table 1. In comparison with their indole-2-carboxamide counterparts 3a and 3b, the 7-azaindole-2-carboxamides 9a and 9b completely lost their binding affinity to the CB_1_ receptor, likely influenced by the poor solubility of these compounds. In contrast, the 6-azaindole-2-carboxamides 3c and 3d exhibited modest binding affinity to the CB_1_ receptor. Interestingly, 6-azaindole-2-carboxamide 3d showed comparable allosteric modulation on the binding of orthosteric agonist CP55,940, while its binding to the CB_1_ receptor was reduced by about 25-fold in comparison with its indole-2-carboxamide counterpart 3b. These results from 9a and 9b suggested that the 7-azaindole is not an optimal bioisostere for replacing the indole ring in the class of CB_1_ allosteric modulators, although it has been successfully used as an effective bioisostere in other indole-containing bioactive molecules.^14^

**Table 1.** Binding Parameters for the Synthesized Compounds

Entry	Compd Code	Structure	K_B_ (μM)^a^	α^b^
3a	LDK1322		0.3	4.2
3b	LDK1326		0.2	5.1
3c	LDK1314		2.4	3.2
3d	LDK1316		5.6	4.4
9a	LDK1313		NB^c^	NA^d^
9b	LDK1315		NB^c^	NA^d^

The red color indicates that the N is the newly introduced nitrogen on the original indole ring.

^a^ K_B_: equilibrium dissociation constant.

^b^ α: binding cooperativity factor.

^c^ NB: no detectable modulation.

^d^ NA: not applicable.

**FIG. 3.**
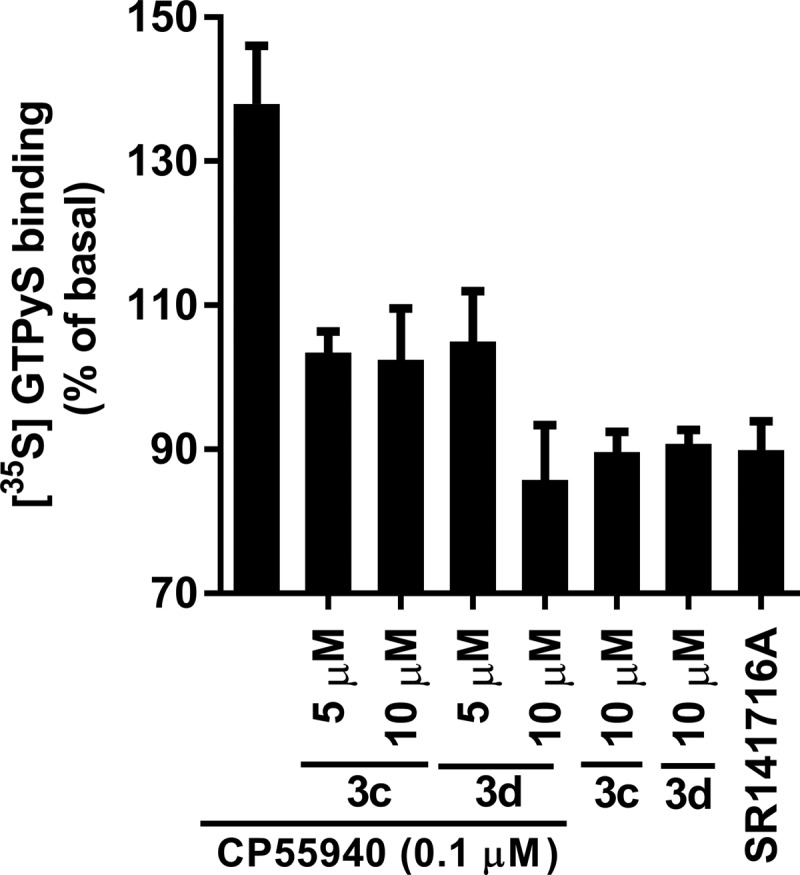
Response of 3c (LDK1314) and 3 d (LDK1316) on [^35^S]GTPγS binding to membranes expressing CB_1_. The effects of 0.1 μM CP55,940 alone, or 0.1 μM CP55,940 with the allosteric modulators 3c or 3d, 3c, or 3d alone, or 1.0 μM SR141716A alone on [^35^S]GTPγS binding were measured at the concentrations indicated. Data are presented as a percentage of basal levels of [^35^S]GTPγS binding. Nonspecific binding was measured in the presence of 10 μM unlabeled GTPγS. Each data point represents the mean±standard error of the mean of at least three independent experiments performed in duplicate. GTPγS, guanosine 5′-O-[gamma-thio]triphosphate.

To investigate G-protein coupling induced by the allosteric modulators, the 6-azaindole-2-carboxamides 3c and 3d were tested for their impact on GTPγS binding. These results, plotted as percent of basal levels of CB_1_ activity, are shown in Figure 3. Both 3c and 3d showed an inhibition of GTPγS binding at 5 and 10 μM in the presence of 0.1 μM CP55,940, compared to membranes treated with 0.1 μM of CP55,940 alone. These data suggest that although the allosteric modulators apparently enhanced the binding of CP55,940, the G-protein coupling induced by CP55,940 was inhibited. In addition, inhibition of G-protein coupling was also seen when CB_1_ was treated with 3c and 3d alone. The level of G-protein coupling inhibition achievable is comparable to that observed with the inverse agonist SR141716A, although the orthosteric compound SR141716A and the allosteric modulators, 3c and 3d, likely inhibit G-protein coupling via different mechanisms.

In the solubility tests, we selected compounds 3c, 3d, and 9a because they represented the indole, 6-azaindole and 7-azaindole scaffold, respectively. We found that compound 9a has exceptionally low solubility in common organic solvents suitable for HPLC sample preparation such as methanol, acetone, and their mixtures with different stoichiometry. We tried the sampling method reported previously to prepare stock solution of 9d into a 2%–5% DMSO in Acetonitrile solution.^22^ Addition of aqueous PBS media led to precipitation of the compound. This made us unable to determine the standard curve for analysis of 9d. Hence, only compounds 3c and 3d were analyzed. The individual solubility of 3c, 3d, and 9a was also simulated using a program (Chemicalize). The simulated aqueous solubility of these three compounds was reported along with the experimental solubility of 3c, 3d, and 9a in Table 2.

**Table 2.** Calculated Aqueous Solubility and the Experimental Thermodynamic Solubility of Compounds 3a, 3c, and 9a

Compound	Aqueous solubility^a^	Thermodynamic solubility^b^
3a	3.0 μg/mL	0 μg/mL
3c	8.0 μg/mL	1.6 μg/mL
9a	6.0 μg/mL	3.0 μg/mL

^a^ Aqueous solubility simulated by computation.

^b^ Thermodynamic solubility obtained from experiments.

The results from solubility studies showed that using 6- and 7-azaindole in lieu of the indole moiety led to enhancement of aqueous solubility compared to the indole counterpart although their solubility are still poor and far below optimal solubility. This finding is in agreement with the results in an early investigation, of which 6- and 7-azaindole analogs showed solubility enhancement in comparison with indole counterpart.^16^ The binding parameters of the synthesized compounds suggested that the 6-azaindole scaffold is worth further exploration, whereas the 7-azaindole ring is not a viable bioisostere of the indole ring in the Org27569 class of CB_1_ allosteric modulators.

Although their binding affinity for CB_1_ was less than their indole-2-carboxamide counterparts, they showed similar coupling characteristics as Org27569, in which they decreased G-protein coupling when compared with treatment with CP55,940 alone, indicating that they may induce signaling in a way similar to Org27569 and its analogs. Further research is needed to fully elucidate the signaling pathways of these compounds; however, it might involve beta-arrestin coupling and signaling such as Org27569 and its previously tested analogs.^20,27,28^ We postulated that the reduced pharmacologic effects of the 6-azaindole-2-carboxamides in comparison with their indole counterparts are likely due to the impact of its electron-deficient pyridine ring on the aromatic π-π stacking during interaction with the allosteric binding site. Therefore, hypothetically, introduction of strong electron-donating groups such as alkoxyl, amino and alkylamino groups on the 6-azaindole moiety may help to improve the pharmacologic effects and aqueous solubility of future compounds bearing the 6-azaindole moiety.

## Supplementary Material

Supplemental data

## Abbreviations Used

ADME: absorption, distribution, metabolism, and excretion
BOP: (benzotriazol-1-yloxy)tris(dimethylamino)phosphonium hexafluorophosphate
BSA: bovine serum albumin
CB_1_: cannabinoid receptor 1
DMSO: dimethylsulfoxide
DMTMM: 4-(4,6-Dimethoxy-1,3,5-triazin-2-yl)-4-methylmorpholinium chloride
EGTA: ethylene glycol-bis(β-aminoethyl ether)-*N*,*N*,*N′*,*N′*-tetraacetic acid
GTPγS: guanosine 5′-O-[gamma-thio]triphosphate
HCl: hydrochloric acid
HOBT: 1-Hydroxybenzotriazole hydrate
MgCl_2_: magnesium chloride
NaCl: sodium chloride
NMM: *N*-methyl morpholine
PBS: phosphate-buffered saline
SAR: structure–activity relationship
THF: tetrahydrofuran
